# Effect of Different Packaging Strategies on the Secondary Shelf Life of Young and Structured Red Wine

**DOI:** 10.3390/foods12142719

**Published:** 2023-07-16

**Authors:** Alessandro Bianchi, Isabella Taglieri, Monica Macaluso, Chiara Sanmartin, Angela Zinnai, Francesca Venturi

**Affiliations:** 1 Department of Agriculture, Food and Environment, University of Pisa, Via del Borghetto 80, 56124 Pisa, Italy; alessandro.bianchi@phd.unipi.it (A.B.); isabella.taglieri@unipi.it (I.T.); monica.macaluso@phd.unipi.it (M.M.); angela.zinnai@unipi.it (A.Z.); francesca.venturi@unipi.it (F.V.); 2 Interdepartmental Research Centre “Nutraceuticals and Food for Health”, University of Pisa, Via del Borghetto 80, 56124 Pisa, Italy

**Keywords:** red wine, sulfur dioxide, capping systems, sensory analysis, screw cap, Tetra Brik, polymeric cap, natural cork, crow cap

## Abstract

When bottled wine is opened, a completely different scenario occurs that can accelerate the oxidation of the product. This is called the secondary shelf life (SSL), which is generally shorter and less predictable than the primary shelf life (PSL). In this context, the research aim was to evaluate the changes that occur in two types of red wine during two tests to evaluate the secondary shelf life as a function of the packaging systems. The variation of Total SO_2_ and Free SO_2_ and the other chemical parameters (polyphenols, anthocyanins, proanthocyanidins, color, and volatile acidity) were used to assess the oxidation rate of the packaging samples after opening during the SSL. In both tests and for the two types of stored red wine, the polymeric cap showed the best results. The other types of closure (screw cap, natural cork, crow cap, and Tetra Brik) showed a negative trend and a reduced SSL for both red wines. Finally, the sensory results confirmed that with the polymeric cap, the SSL increases considerably compared to other capping systems. These results may be due to the technical characteristics of polymeric materials, which tend to vary slightly in shape after repeated usage.

## 1. Introduction

In general, the shelf-life of food products can be divided into two categories: (i) the primary shelf life (PSL), defined as a finite length of time after packaging during which the product retains an established quality level under well-defined storage conditions and (ii) the secondary shelf life (SSL), defined as the period after opening the package during which a food maintains its characteristics if stored under the conditions indicated on the label [1,2,3].

The secondary shelf life starts with the opening of the food package, which determines a sharp change in its environmental conditions (composition of the atmosphere, greater presence of oxygen, humidity, temperature fluctuations, loss of sterility, etc.), leading to a strong acceleration of the quality decay rate of the product [3,4,5,6,7,8]. Since the opening of the package may occur at different times along the primary shelf life, the quality of the product when the package is opened could be highly variable, making it really complicated to predict the final length of the secondary shelf life [1,3,9].

Nevertheless, a definition of the best operating conditions to be adopted to maximally extend the secondary shelf life of foods could provide a valid strategy for the reduction of waste not only at an industrial level but also for domestic use [1,9,10].

In this context, bottled wine follows very particular dynamics that place it outside the complex panorama of food products. On the label, in fact, there is no indication regarding the expiration date, and the definition of the end point of the PSL remains mostly entrusted to the sensitivity of the producer and/or consumer [11,12].

Gaseous atmosphere in the headspace, type of packaging, light conditions, and temperature play a fundamental role in defining the evolution of the quality of the wine during storage, as long as the package is properly closed [4,7,13,14,15,16]. The opening of the bottle represents a “traumatic” event because it accelerates the degradation phenomenon as a consequence of the uncontrolled access of oxygen inside the package, altering the quality of the product [1,17,18]. As a function of the chemical composition of the specific wine, the choice of the type of package will have a considerable impact on wine oxidation, and therefore, it will affect its chemical and sensory properties [16,19,20,21,22,23,24]. The classical packaging material for wine is glass and a natural cork stopper [25]. During the past two decades, however, other materials have been used for the packaging and closure of wine, including polyethylene terephthalate (PET) bottles, polymeric caps, screw caps, crown caps, multilayer Tetra Brik, and bag-in-box type containers [14,17,18,25,26,27].

Despite numerous studies aimed at understanding the evolution of PSL in different storage and packaging conditions [14,21,28,29,30], the scientific literature is certainly lacking in information useful for understanding the mechanism of wine’s SSL. Furthermore, especially in Italy, among the most common habits in the daily lives of many consumers is the prolonged consumption of bottled wine over time, either for consumption with meals or as an ingredient in cooking [10,31].

Given the increasingly strong health claims associated with the consumption of alcoholic beverages, domestic consumption of a bottle of wine divided over several occasions is very common [1,9,10]. For this reason, the aim of this work is to evaluate the changes that take place in young and structured red wines during their SSL for different packaging materials and capping systems. It is also of interest to understand the role played by the different closing systems of the bottles when they are subjected to repeated cycles of opening and closing.

## 2. Materials and Methods

### 2.1. Experimental Design

As reported in a previous study related to white wine [1], two different simulation tests were carried out for the evaluation of the secondary shelf life of 2 types (young (Y) and structured (S)) of commercial red wine:

- Test 1 (static way—without air exchange during storage after first opening): at the beginning 20 bottles for each type of package were opened, half emptied (removal of 375 mL of wine), manually closed with the same cap, and without further replacement of the gaseous atmosphere in the headspace during the whole observation time. Four containers of each type were analyzed in correspondence with each sampling time (0, 3, 7, 15, and 30 days). To avoid complete damage to the caps, the corkscrew was inserted up to half of their length;
- Test 2 (dynamic way—with air exchange during storage after first opening): a total of 20 containers for each type of package were repeatedly opened and closed manually with the same cap in correspondence with predetermined time intervals (0, 3, 7, 15, and 30 days). At each opening cycle, 75 mL of wine was removed from each package, and then the package was manually closed with the same cap until the next wine opening. To avoid complete damage to the caps, the corkscrew was inserted up to half of their length.

In both tests, the same 2 types (young (Y) and structured (S)) of red wine were packaged in the different packaging/capping systems (Figure S1) at the same time as reported in Table 1 and stored at a controlled temperature (15 °C) during the observation period. The chemical parameters of 2 types of red wine at time 0 are reported in Table S1.

### 2.2. Chemical Analysis

The chemical characterization of red wine (Titratable acidity (TTA) (tartaric acid g/L), pH, alcohol content (%*v/v*), Volatile acidity (VA) (g/L acetic acid), Total polyphenols (TPP) (g/L gallic acid), Proanthocyanidins (PA) (mg/L catechins), Total anthocyanins (TA) (mg/L malvidin), Color Intensity (CI), and Tonality (T) were carried out according to the OIV methods as previously described [32]. Total SO_2_ (TSO_2_) (mg/L) and Free SO_2_ (FSO_2_) (mg/L) were carried as previously described [33]. The time evolution of sulfur dioxide concentration (TSO_2_ and FSO_2_) has also been described by a first-order kinetic equation, as previously reported [14]. In fact, the kinetic constants (k) may be considered a valid measure of the effect induced by oxidation during wine storage as a function of the packaging strategies.

### 2.3. Sensory Analysis

The sensory panel of the Department of Agriculture, Food, and Environment, comprised of 10 certified panelists (4 males and 6 females, 23–60 years), selected and trained as described [34], performed the sensory analysis, following the sensory sheet described by Bianchi et al., 2022 [35], with the same modifications to be used in the SSL evaluation of wine. In particular, the sensory sheet includes quantitative parameters (color intensity, oxidation, absence of defects, acidity, softness, astringency, aftertaste, balance, and aging degree) and hedonic parameters (visual attractiveness, olfactory pleasantness, tasting pleasantness, and global pleasantness).

The overall hedonic index (HI) of red wine during the secondary shelf life was calculated as the mean of the hedonic parameters converted on a scale from 0 to 10 according to Bianchi et al., 2022 [29].

### 2.4. Statistical Analysis

The analyses of the data were carried out with the JMP Pro 17 software package (SAS Institute, Cary, NC, USA). To evaluate the statistical significance of the experimental data, each sample was analyzed in quadruplicate. The change in chemical parameters was expressed as a percentage compared to the initial value.

One Way Completely Randomized ANOVA (CoStat, version 6.451, CoHort Software, Pacific Grove, CA, USA) was applied to evaluate the reliability of the data sets. Tukey’s HSD multiple mean comparison test (*p* ≤ 0.05) was used to state the differences among samples.

Sensory analysis results were processed by Big Sensory Soft 2.0 (version 2018, Centro Studi e Formazione Assaggiatori, Brescia, Italy). In particular, sensory data were analyzed by a two-way ANOVA with panelists and samples as the main factors. The hierarchical cluster analysis (HCA) based on the quantitative value was performed applying the Ward method and using two-way clustering.

## 3. Results and Discussion

### 3.1. Test 1—Without Air Exchange

Test 1 represents the condition that simulates the domestic reuse of open bottles of red wine many days after their first opening. During the test, it was possible to verify the reaction of the different closing systems to the stress of opening and their residual ability to maintain oxygen impermeability after the first deformation.

In Figures S2a,b and S3a,b the decrease of both TSO_2_ and FSO_2_ at each sampling time is reported. According to previously published results [1,14,25], the reduction in concentration of TSO_2_ and FSO_2_ can be related to the oxidation rate of the stored samples. In order to highlight the different barrier effects of the tested packaging strategies, the kinetic constants of the degradation of sulfur dioxide were calculated according to a first-order kinetic [14].

As reported in the literature [1,14,25], the degradation rate of the TSO_2_ could be affected both by the packaging systems and by the polyphenol content of the wine. According to our results (Table 2), the structured wine has a longer SSL than the young wine, as expected considering their polyphenol content. Moreover, the packaging strategy strongly affected the TSO_2_ and FSO_2_ degradation rates: the fastest kinetic was observed in the natural cork, Tetra Brik, and crown cap, while the best performance was recorded for the wine closed with the polymeric cap. Therefore, among all the tested conditions, the polymeric cap allowed the longest SSL regardless of the tested wine, with a kinetic constant always lower than half of the others.

The other parameters related to the decrease in shelf life of products are the negative variation in TTP, PA, TA, and CI and the increase in VA and T [1,19].

As reported in Table 3, taking into account the variation of all the considered parameters, after 30 days of storage, higher oxidation rates were observed in the samples closed by crown caps (Y-C and S-C) and in Tetra Brik containers (Y-TB and S-TB), closely followed by natural cork stoppers (Y-N and S-N) and screw caps (Y-S and S-S).

All these closures were found to be significantly deformed already after the first opening, to the extent that they lost their barrier effect against the external atmosphere. On the contrary, the polymeric cap allowed the best results in terms of the wine’s secondary shelf life because, despite the initial stress corresponding to the first opening, it appeared to still be able to completely recover its initial shape when the bottle was closed again.

According to our results, the dense polymer network of the synthetic cap, which could represent a limitation for its use during prolonged wine storage [13,14,15,36], seems to be an advantage when we consider the SSL of red wine.

Further, while the results showed that the two types of red wine had the same trend in all parameters analyzed, it was possible to highlight that the young wine had a statistically higher percentage variation than the structured red wine. This can be related [37,38,39,40] to the lower antioxidant compound content (polyphenols, anthocyanins, and proanthocyanins) present in the young wine compared to the structured one (Table S1).

### 3.2. Test 2—With Air Exchange

Test 2 aimed to verify the reaction of the different closing systems to the stress of opening and reuse in repeated cycles, simulating the daily domestic use of wine over time.

During the first days (3–7 days) of SSL, the decrease in concentration of the main chemical parameters was lower than that observed in test 1, regardless of the type of closure considered (Tables S2 and S3). This can be explained by the lower availability of air in the headspace of the container, which could slow down the wine’s oxidation rate, at least for the first half of the entire observation period. In fact, considering that the percentage of O_2_ in the atmosphere is about 20.95%, during the first packaging opening, 78.7 mL of O_2_ were introduced in test 1, while only 15.7 mL of O_2_ were introduced in test 2.

On the contrary, at the end of the observation period, the decrease in TSO_2_ (Figures S4a and S5a) and FSO_2_ (Figures S4b and S5b) was higher than that observed in test 1, regardless of the type of closure considered. This can be due to the repeated deformation of the closure, which allows a greater gas exchange in the headspace of each bottle used in test 2. This is also confirmed by the kinetic constants of sulfur dioxide degradation calculated for test 2 (Table 4), which are twofold compared to test 1, demonstrating that repeated opening cycles caused an increase in the rate of quality depletion, but the trend remained the same. Additionally, in this case, the worst performances were observed for the crown, strictly followed by natural cork and Tetra Brik, while the best one was always the polymeric cap. The screw cap showed an intermediate k value compared to the others, and, contrary to expectations, it was not able to preserve the wine from oxidation.

If we consider the SO_2_ percentage decrease after 30 days from the first opening, the polymeric cap led to a smaller decrease than the other closures, and in addition, the residual content of FSO_2_ was not enough to preserve the quality of red wine. According to what was observed for test 1, in test 2, the polymeric cap still maintains its structural characteristics better than other types of closure, even after repeated opening/closing operations. The higher retention effect of TSO_2_ and FSO_2_ was also confirmed by a lower percentage increase in VA and T and a lower decrease in TPP, TA, PA, and CI (Table 5) in wine samples closed with the polymeric cap (Y-P and S-P) compared to all others.

These results may be due to the technical characteristics of polymeric material that tend to vary its shape to a limited extent even after repeated solicitations (low susceptibility to deformations, lacerations, oxygen tightness, etc.) [1].

The screw cap showed problems related to the pressure it exerts on the mouth of the bottle following the first opening because, once removed from the ring, even if screwed with force, it does not seem to completely recover the original sealing properties.

The cork was found to be less durable than polymerics as it cracks and/or crumbles easily due to repeated opening, compromising the overall seal as a result of repeated stresses.

The crown cap, if it deforms at the time of opening, no longer adheres optimally to the mouth of the bottle when used subsequently, losing in fact its protection capabilities in front of oxygen.

### 3.3. Sensory Evaluation

The hedonic quality level of a product is fundamental in determining its acceptability and overall pleasantness.

**Figure 1:**
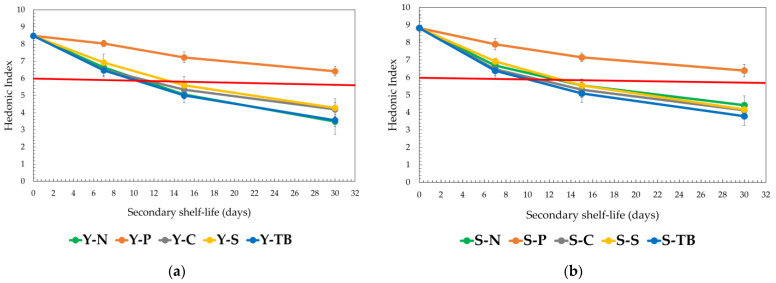
Trend of hedonic index (HI) during the period in the test without air exchange (test 1). The data are expressed as mean ± SD. The red line indicates the HI reference limit of SSL (HI = 6): (**a**) young red wine and (**b**) structured red wine.

The overall hedonic index (HI) of samples for test 1 is reported in Figure 1a,b; a value of 6 out of 10 was taken as a reference point for the acceptability limit to be considered for ending the SSL of red wine. The end of the sensory acceptability of samples was strongly affected by the packaging systems adopted, and the sensory results confirmed what was observed for the evolution of chemical parameters. Indeed, the SSL of both red wines closed with polymeric caps (Y-P and S-P) was the highest, keeping its HI ≥ 6 even after 30 days of storage. Instead, both red wines closed by the other capping systems tested (natural cork, screw cap, crown cap, and Tetra Brik) showed a shorter SSL after just 10 days from the opening; they were no longer acceptable (HI < 6).

**Figure 2:**
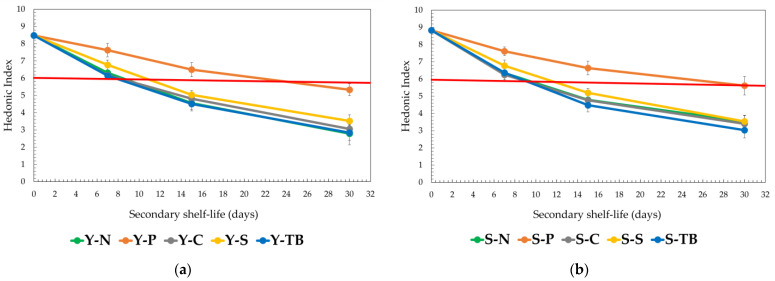
Trend of hedonic index (HI) during the period in the test with air exchange (test 2). The data are expressed as mean ± SD. The red line indicates the HI reference limit of SSL: (**a**) young red wine and (**b**) structured red wine.

If we analyze the evolution of the HI (Figure 2a,b) for test 2, we can observe a faster decrease in the values. This is probably due to the repeated cycles of opening/closing, which lead to a faster degradation of the product, and this is evident just from the second cycle (7 days). Anyway, the polymeric caps (Y-P and S-P) showed the best results compared with the others in this trial, but the SSL is lower (15 days) than test 1. For the other packaging systems, the SSL is thus reduced to 7 days.

If we analyze the two-way HCA of the quantitative parameters (Figure S6a,b) of test 1, the samples are grouped together according to the same trend highlighted by the chemical data. In fact, the young and structured wines closed with the polymeric cap (Y-P-7 and S-P-7) after 7 days show a behavior statistically different from the other types of closure. Further, polymeric caps showed the best performance at each sampling time.

For test 2, the HCA (Figure S7a,b) shows different associations, and according to the chemical data, in the first 7 days, the samples tend to differentiate from time 0 and cluster all together. On the contrary, when the storage time increases, all the other packaging systems tend to cluster together according to the sampling times, while the polymeric cap exhibits a slower decay rate of the sensory profile.

In all the HCA analyzed, the six quantitative parameters are grouped into three clusters: (i) color intensity, absence of defects, and acidity; (ii) softness, astringency, and balance; and (iii) aftertaste, oxidation, and aging degree.

As reported in the literature regarding the PSL [6,14,29], these data highlight the weight of the parameters useful to define the SSL of red wine. According to the HCA, the third cluster of parameters (aftertaste, oxidation, and aging degree) are those most suitable to differentiate the samples and the most related to the oxidation and evolution of wine during SSL.

**Figure 3:**
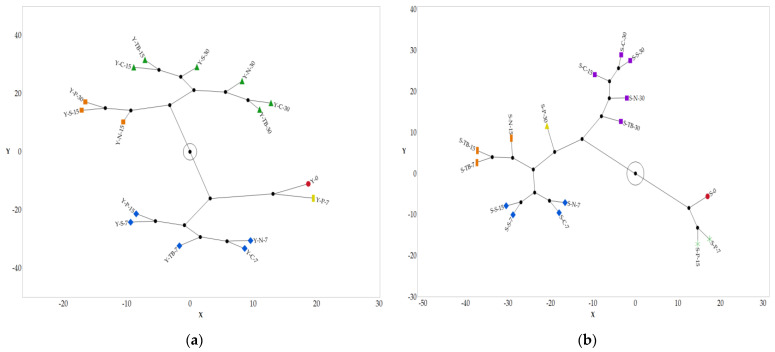
Constellation diagram generated from the HCA for the test without air exchange (test 1). The number next to the code (0-7-15-30) indicates the days of SSL for each sample: (**a**) young red wine and (**b**) structured red wine.

**Figure 4:**
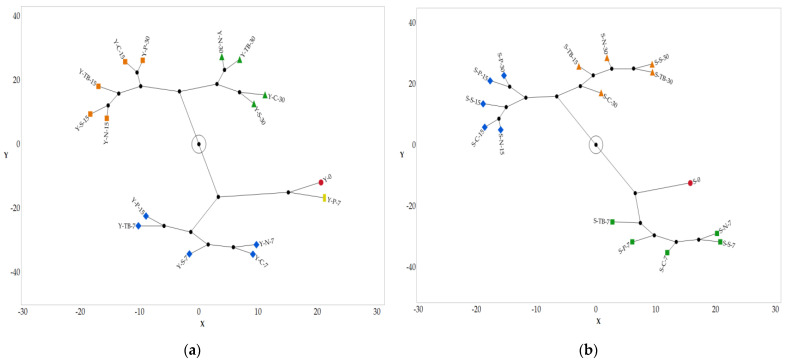
Constellation diagram generated from the HCA for the test with air exchange (test 2). The number next to the code (0-7-15-30) indicates the days of SSL for each sample: (**a**) young red wine and (**b**) structured red wine.

To summarize and better visualize the different families, a constellation diagram was generated from the HCA for test 1 (Figure 3a,b) and for test 2 (Figure 4a,b).

According to the data related to test 1, it is possible to estimate an SSL equal to at least 30 days for both the young and structured wine closed with a polymeric cap, while the other packaging systems maintain their quality above an acceptable limit until 15 days.

Regarding test 2, the estimation of SSL is equal to 15 days for the polymeric cap and less than 7 days for other packaging systems.

These results are in complete agreement with the chemical trend of the main compositional parameters evaluated, showing that the sensory features can be adopted as a suitable marker of wine acceptability.

**Figure 5:**
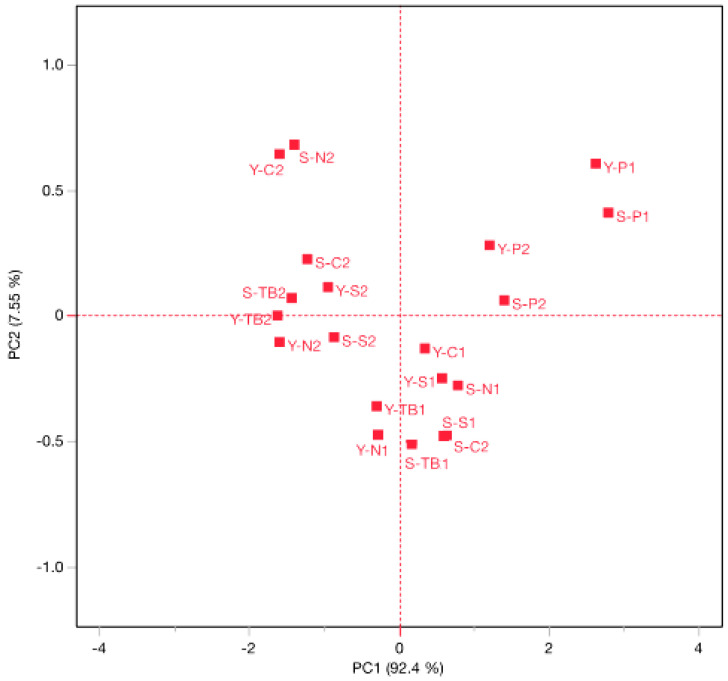
PCA for the correlation between the hedonic index (HI) and kinetic constants of total sulfur dioxide (k_TSO2_). The numbers 1 and 2 in the code indicate tests 1 and 2, respectively.

In particular, the hedonic index (HI) showed a strong negative correlation (−0.85) with the kinetic constants related to the degradation of total sulfur dioxide (k_TSO2_), as highlighted by the PCA graph reported in Figure 5. The samples can be separated as a function of the tests (1 and 2); moreover, the samples with the polymeric cap behaved in a distinctive way, grouping together.

## 4. Conclusions

Wine is a complex matrix, and its secondary shelf life is governed by numerous factors related to the wine itself, the storage conditions, and above all, the packaging system.

The results obtained suggest that the sulfur dioxide degradation constant as well as its sensory decay are representative indexes of the quality depletion of wine during home use.

The polymeric stopper has proved to be a valid tool during home wine storage because it tends to vary limitedly in its shape, even after repeated usage, as highlighted by the low TSO_2_ degradation rate and the good sensory acceptability in both tests, regardless of the type of wine (young and structured).

On the contrary, the natural cork stopper, which generally represents the reference system for bottling wine during its primary shelf life, has not proved to be entirely suitable for a secondary shelf-life study. In test 1 and especially in test 2, the natural cork stopper was shown to be strongly affected by the physical stress received in opening/closing cycles, losing its initial features.

The crown cap has shown critical issues in SSL tests, both in static and dynamic approaches. The deformation that occurs at the first opening greatly compromises its “tightness” characteristics against the inlet of air from the outside.

Finally, the screw cap and the Tetra Brik, much touted for their effectiveness in sealing packages intended for time-delayed use, did not seem to have confirmed their good reputation during the presented secondary shelf-life tests.

Although these results appear to be in stark contrast to what is reported in the literature regarding wine PSL studies [4,13,14,15], they are in perfect agreement with those recently reported for SSL of white wine [1].

## Figures and Tables

**Table 1:** Sample codes used in the study based on the different combinations of packaging/capping systems.

Packaging System	Cap Size (Diameter × Height)	Container Volume	Sample Code Young Red Wine	Sample Code Structured Red Wine
Glass + Natural cork	(24.0 mm × 45.0 mm)	750 mL	Y-N	S-N
Glass + Polymeric cap	(23.0 mm × 44.0 mm)	750 mL	Y-P	S-P
Glass + Screw cap	(25.6 mm × 33.0 mm)	750 mL	Y-S	S-S
Glass + Crown cap	(26.8 mm × 6.8 mm)	750 mL	Y-C	S-C
Tetra Brik	(25.0 mm × 11.2 mm)	750 mL	Y-TB	S-TB

**Table 2:** Kinetic constants of the degradation of sulfur dioxide (TSO_2_ and FSO_2_) in the test without air exchange (test 1). The data are the mean ± SD for four replicates.

Sample	k_TSO2_ (Days^−1^)	k_FSO2_ (Days^−1^)
Y-N	0.0224 ^ab^	0.0403 ^ab^
Y-P	0.0087 ^d^	0.0200 ^c^
Y-C	0.0203 ^b^	0.0382 ^b^
Y-S	0.0177 ^c^	0.0388 ^b^
Y-TB	0.0234 ^a^	0.0419 ^a^
S-N	0.0159 ^b^	0.0303 ^b^
S-P	0.0060 ^c^	0.0141 ^c^
S-C	0.0158 ^b^	0.0338 ^a^
S-S	0.0154 ^b^	0.0288 ^b^
S-TB	0.0188 ^a^	0.0329 ^a^

Different letters in each column for each group of red wines (Y and S) refer to significant differences (Tukey, *p* ≤ 0.05).

**Table 3:** Percentage variation in the chemical parameters after 30 days in the test without air exchange (test 1). The data are expressed as the mean ± SD for four replicates.

Sample	Variation in TPP (%)	Variation in TA (%)	Variation in PA (%)	Variation in CI (%)	Variation in T (%)	Variation in VA (%)
Y-N	−7.95 ^c^	−16.99 ^b^	−9.84 ^c^	−22.26 ^b^	18.97 ^b^	10.21 ^ab^
Y-P	−3.82 ^d^	−10.65 ^c^	−3.48 ^d^	−15.12 ^c^	10.84 ^c^	5.79 ^c^
Y-C	−9.00 ^b^	−16.97 ^b^	−10.59 ^bc^	−24.00 ^a^	19.90 ^a^	10.53 ^a^
Y-S	−8.18 ^c^	−16.63 ^b^	−10.85 ^b^	−21.01 ^b^	18.29 ^b^	9.78 ^b^
Y-TB	−9.90 ^a^	−18.51 ^a^	−13.44 ^a^	−24.09 ^a^	19.13 ^ab^	10.66 ^a^
S-N	−9.02 ^a^	−16.94 ^a^	−10.58 ^b^	−19.07 ^ab^	16.22 ^ab^	9.14 ^a^
S-P	−4.79 ^c^	−9.74 ^c^	−5.88 ^c^	−10.97 ^c^	9.13 ^c^	4.89 ^c^
S-C	−9.54 ^a^	−15.65 ^ab^	−11.79 ^a^	−19.67 ^a^	16.80 ^a^	8.99 ^ab^
S-S	−7.48 ^b^	−14.17 ^b^	−9.94 ^b^	−18.92 ^b^	15.92 ^b^	8.50 ^b^
S-TB	−9.32 ^a^	−16.16 ^a^	−11.02 ^a^	−19.67 ^a^	16.22 ^ab^	9.82 ^a^

Different letters in each column for each group of red wines (Y and S) refer to significant differences (Tukey, *p* ≤ 0.05).

**Table 4:** Kinetic constant of the degradation of the sulfur dioxide (TSO_2_ and FSO_2_) in the test with air exchange (test 2). The data are the mean ± SD for four replicates.

Sample	k_TSO2_ (Days^−1^)	k_FSO2_ (Days^−1^)
Y-N	0.0350 ^b^	0.0960 ^a^
Y-P	0.0169 ^d^	0.0445 ^c^
Y-C	0.0408 ^a^	0.0944 ^a^
Y-S	0.0318 ^c^	0.0685 ^b^
Y-TB	0.0360 ^b^	0.0945 ^a^
S-N	0.0396 ^a^	0.0693 ^c^
S-P	0.0138 ^d^	0.0304 ^d^
S-C	0.0347 ^b^	0.0838 ^a^
S-S	0.0297 ^c^	0.0696 ^c^
S-TB	0.0351 ^b^	0.0736 ^b^

Different letters in each column for each group of red wines (Y and S) refer to significant differences (Tukey, *p* ≤ 0.05).

**Table 5:** Percent variation in the chemical parameters after 30 days in the test with air exchange (test 2). The data are expressed as the mean ± SD for four replicates.

Sample	Variation in TPP (%)	Variation in TA (%)	Variation in PA (%)	Variation in CI (%)	Variation in T (%)	Variation in VA (%)
Y-N	−17.03 ^b^	−21.70 ^c^	−17.17 ^b^	−20.89 ^b^	15.28 ^b^	17.02 ^b^
Y-P	−8.83 ^d^	−11.31 ^e^	−10.72 ^c^	−10.00 ^d^	7.58 ^c^	8.07 ^d^
Y-C	−18.79 ^a^	−26.09 ^a^	−20.12 ^a^	−22.45 ^a^	17.82 ^a^	16.69 ^a^
Y-S	−17.76 ^b^	−16.79 ^d^	−17.77 ^b^	−17.12 ^c^	14.14 ^b^	15.64 ^c^
Y-TB	−16.56 ^c^	−23.73 ^b^	−20.10 ^a^	−22.44 ^a^	18.36 ^a^	17.98 ^ab^
S-N	−15.86 ^a^	−27.37 ^a^	−14.29 ^b^	−16.63 ^c^	13.46 ^c^	15.59 ^a^
S-P	−6.74 ^d^	−13.64 ^d^	−11.32 ^c^	−7.36 ^d^	5.82 ^d^	5.67 ^c^
S-C	−15.72 ^a^	−25.93 ^b^	−15.93 ^ab^	−17.64 ^bc^	14.84 ^b^	13.12 ^b^
S-S	−13.04 ^c^	−24.48 ^c^	−16.83 ^a^	−16.89 ^c^	13.15 ^c^	12.79 ^b^
S-TB	−14.88 ^b^	−24.88 ^bc^	−17.42 ^a^	−18.65 ^a^	16.80 ^a^	15.92 ^a^

Different letters in each column for each group of red wines (Y and S) refer to significant differences (Tukey, *p* ≤ 0.05).

## Data Availability

Data are contained within the article.

## Supplementary Materials

The following supporting information can be downloaded at: https://www.mdpi.com/article/10.3390/foods12142719/s1, Figure S1: Image of the capping systems used in the trials; Figure S2: Percentage decrease of SO_2_ in young red wine (Y) during the secondary shelf-life (3-7-15-30 days) in the test without air exchange (test 1). The data are expressed as the mean ± SD for four replicates. Different letters in each group refer to significant differences (Tukey, *p* ≤ 0.05): (a) Total SO_2_; (b) Free SO_2_; Figure S3: Percentage decrease of SO_2_ in structured red wine (S) during the Secondary shelf life (3-7-15-30 days) in the test without air exchange (test 1). The data are expressed as the mean ± SD for four replicates. Different letters in each group refer to significant differences (Tukey, *p* ≤ 0.05): (a) Total SO_2_; (b) Free SO_2_; Figure S4: Percentage decrease of SO_2_ in young red wine (Y) during the Secondary shelf-life (3-7-15-30 days) in the test with air exchange (test 2). The data are expressed as the mean ± SD for four replicates. Different letters in each group refer to significant differences (Tukey, *p* ≤ 0.05): (a) Total SO_2_; (b) Free SO_2_; Figure S5: Percentage decrease of SO2 in structured red wine (S) during the Secondary shelf-life (3-7-15-30 days) in the test with air exchange (test 2). The data are expressed as the mean ± SD for four replicates. Different letters in each group refer to significant differences (Tukey, *p* ≤ 0.05): (a) Total SO_2_; (b) Free SO_2_; Figure S6: Hierarchical cluster analysis (HCA) based on quantitative data in the test without air exchange (test 1). The number next to the code (0-7-15-30) indicates the days of SSL for each sample: (a) young red wine and (b) structured red wine; Figure S7: Hierarchical cluster analysis (HCA) based on quantitative data in the test with air exchange (test 2). The number next to the code (0-7-15-30) indicates the days of SSL for each sample: (a) young red wine and (b) structured red wine; Table S1: Chemical parameters of red wine used during the research. The data are expressed as the mean ± SD for four replicates.; Table S2: Percentage variation in the chemical parameters during the secondary shelf-life (3-7-15-30 days) in the test without air exchange (test 1). The data are expressed as the mean ± SD for four replicates; Table S3: Percentage variation in the chemical parameters during the secondary shelf-life (3-7-15-30 days) in the test with air exchange (test 2). The data are expressed as the mean ± SD for four replicates.
