# Supplementary material for: Effect of Different Packaging Strategies on the Secondary Shelf Life of Young and Structured Red Wine
Source: Foods. 2023 Jul 16;12(14):2719. doi: 10.3390/foods12142719 (PMC10379816; doi:10.3390/foods12142719)
Supplement: Supplementary file 1 [file foods-12-02719-s001.zip › foods-2474693-supplementary.pdf]

Table S1. Chemical parameters of red wine used during the research. Data are expressed as mean ± SD for 4 replicates.

Parameters	Units	Young red wine (Y)	Structured red wine (S)
pH		3.45 ± 0.03	3.51 ± 0.03
Titratable acidity (TTA)	g/L tartaric acid	4.96 ± 0.14	4.26 ± 0.12
Volatile acidity (VA)	g/L acetic acid	0.46 ± 0.04	0.61 ± 0.04
Alcohol content	%V/V	12.5 ± 0.3	14.0 ± 0.3
Total polyphenols (TPP)	g/L gallic acid	1.23 ± 0.02	2.53 ± 0.02
Total anthocyanins (TA)	mg/L malvidin	370 ± 5	608 ± 7
Proanthocyanidins (PA)	mg/L catechins	813 ± 4	1405 ± 9
Color Intensity (CI)	-	1.29 ± 0.04	0.62 ± 0.03
Tonality (T)	-	0.99 ±0.05	0.74 ± 0.03
Total SO_2 (TSO_2)	mg/L	98 ± 2	95 ± 2
Free SO_2 (FSO_2)	mg/L	19 ± 1	22 ± 1

Table S2. Percentage variation in the chemical parameters during the Secondary shelf-life (3-7-15-30 days) in the test without air exchange (test 1). Data are expressed as mean ± SD for 4 replicates.

Sample	SSL (days)	Variation in TTP (%)	Variation in TA (%)	Variation in PA (%)	Variation in CI (%)	Variation in T (%)	Variation in VA (%)
Y-N	3	-2.39 ± 0.14	-4.14 ± 0.14	-3.11 ± 0.21	-4.72 ± 0.14	2.97± 0.21	3.16 ± 0.19
	7	-4.23 ± 0.26	-8.87 ± 0.17	-4.76 ± 0.29	-9.31 ± 0.17	5.95 ± 0.29	6.38 ± 0.13
	15	-6.31 ± 0.17	-12.85 ± 0.16	-7.25 ± 0.34	-17.63 ± 0.16	10.62 ± 0.34	8.43 ± 0.25
	30	-7.95 ± 0.34	-16.99 ± 0.27	-9.84 ± 0.31	-20.89 ± 0.27	15.28 ± 0.31	10.21 ± 0.24
Y-P	3	-1.28 ± 0.27	-2.81 ± 0.33	-1.26 ± 0.27	-3.29 ± 0.33	1.42 ± 0.27	1.36 ± 0.17
	7	-1.89 ± 0.17	-3.20 ± 0.13	-1.86 ± 0.33	-6.21 ± 0.13	2.74 ± 0.33	2.63 ± 0.16
	15	-2.26 ± 0.22	-7.89 ± 0.45	-2.86 ± 0.34	-8.21 ± 0.45	5.72 ± 0.34	3.83 ± 0.12
	30	-3.82 ± 0.31	-10.65 ± 0.32	-3.48 ± 0.22	-10.00 ± 0.32	7.58 ± 0.22	5.79 ± 0.23
Y-C	3	-3.45 ± 0.21	-5.57 ± 0.17	-3.41 ± 0.37	-5.79 ± 0.17	3.31 ± 0.37	3.16 ± 0.17
	7	-4.84 ± 0.27	-8.48 ± 0.37	-5.06 ± 0.31	-10.09± 0.37	7.49 ± 0.31	5.31 ± 0.25
	15	-6.94 ± 0.39	-11.87 ± 0.24	-7.85 ± 0.47	-18.61 ± 0.24	12.16 ± 0.47	7.99 ± 0.19
	30	-9.00 ± 0.45	-16.97 ± 0.35	-10.59 ± 0.17	-22.45 ± 0.35	17.82 ± 0.17	10.53 ± 0.43
Y-S	3	-3.41 ± 0.53	-3.47 ± 0.23	-3.81 ± 0.20	-6.40 ± 0.23	2.06 ± 0.20	2.65 ± 0.21
	7	-4.04 ± 0.35	-6.66 ± 0.16	-5.44 ± 0.31	-10.10 ± 0.36	4.38 ± 0.31	4.33 ± 0.29
	15	-6.44 ± 0.39	-12.98 ± 0.45	-8.52 ± 0.30	-14.01 ± 0.45	9.08 ± 0.30	7.73 ± 0.41
	30	-8.18 ± 0.46	-16.63 ± 0.22	-10.85 ± 0.29	-17.12 ± 0.22	14.14 ± 0.29	9.78 ± 0.48
Y-TB	3	-2.60 ± 0.23	-4.46 ± 0.23	-3.71 ± 0.22	-5.41 ± 0.23	3.46 ± 0.22	2.32 ± 0.16
	7	-4.73 ± 0.21	-9.18 ± 0.26	-5.53 ± 0.11	-6.97 ± 0.26	7.95 ± 0.21	5.46 ± 0.21
	15	-5.82 ± 0.29	-13.68 ± 0.35	-7.86 ± 0.24	-17.83 ± 0.35	13.70 ± 0.34	7.45 ± 0.29
	30	-9.90 ± 0.36	-18.51 ± 0.31	-13.44 ± 0.26	-22.44 ± 0.31	18.36 ± 0.36	10.66 ± 0.36
S-N	3	-2.03 ± 0.17	-6.10 ± 0.18	-3.38 ± 0.19	-2.58 ± 0.33	2.09 ± 0.27	3.54 ± 0.17
	7	-3.07 ± 0.14	-10.53 ± 0.26	-5.09 ± 0.26	-8.85 ± 0.23	4.95 ± 0.33	5.70 ± 0.31
	15	-5.19 ± 0.14	-13.32 ± 0.23	-7.66 ± 0.32	-7.89 ± 0.45	8.53 ± 0.34	7.02 ± 0.31
	30	-9.02 ± 0.43	-16.94 ± 0.34	-10.58 ± 0.33	-16.63 ± 0.52	13.46 ± 0.42	9.14 ± 0.23
S-P	3	-1.00 ± 0.12	-2.43 ± 0.16	-1.05 ± 0.27	-1.67 ± 0.16	0.78 ± 0.27	1.08 ± 0.33
	7	-1.96 ± 0.22	-3.32 ± 0.27	-3.10 ± 0.21	-3.09 ± 0.27	2.82 ± 0.21	2.29 ± 0.12
	15	-2.35 ± 0.33	-6.45 ± 0.22	-4.24 ± 0.39	-4.70 ± 0.22	4.92 ± 0.39	3.06 ± 0.43
	30	-4.79 ± 0.26	-9.74 ± 0.31	-5.88 ± 0.45	-7.36 ± 0.31	5.82 ± 0.45	4.89 ± 0.26
S-C	3	-2.43 ± 0.13	-5.23 ± 0.28	-3.03 ± 0.25	-3.09 ± 0.18	2.69 ± 0.35	4.05 ± 0.35
	7	-3.95 ± 0.17	-7.94 ± 0.21	-5.33 ± 0.27	-7.50 ± 0.31	5.37 ± 0.37	5.40 ± 0.31
	15	-6.98 ± 0.25	-11.38 ± 0.29	-8.23 ± 0.29	-11.08 ± 0.29	9.91 ± 0.29	7.22 ± 0.21
	30	-9.54 ± 0.24	-15.65 ± 0.36	-11.79 ± 0.23	-17.64 ± 0.46	14.84 ± 0.43	8.99 ± 0.34
S-S	3	-2.80 ± 0.13	-4.42 ± 0.23	-4.02 ± 0.25	-3.19± 0.23	2.02 ±0.25	2.40 ± 0.20
	7	-4.02 ± 0.35	-7.62 ± 0.35	-6.70 ± 0.31	-7.84 ± 0.45	5.20 ± 0.21	4.05 ± 0.25
	15	-5.80 ± 0.25	-11.71 ± 0.39	-8.02 ± 0.29	-10.77 ± 0.29	8.95 ± 0.39	6.95 ± 0.35
	30	-7.48 ±0.22	-14.17 ± 0.46	-9.94 ± 0.38	-16.89 ± 0.56	13.15 ±0.48	8.50 ± 0.12
S-TB	3	-2.48 ± 0.13	-6.13 ± 0.29	-4.43 ± 0.16	-3.59 ± 0.29	3.65 ± 0.16	3.49 ± 0.11
	7	-3.58 ± 0.15	-9.91 ± 0.15	-6.33 ± 0.20	-7.91 ± 0.15	6.75 ± 0.20	6.43 ± 0.25
	15	-5.17 ± 0.36	-14.47 ± 0.21	-9.40 ± 0.24	-12.90 ± 0.21	10.47 ± 0.24	8.32 ± 0.16
	30	-9.32 ± 0.38	-16.16 ± 0.41	-11.02 ± 0.34	-18.65 ± 0.41	16.80 ± 0.34	9.82 ± 0.28

Table S3. Percentage variation in the chemical parameters during the Secondary shelf-life (3-7-15-30 days) in the test with air exchange (test 2). Data are expressed as mean ± SD for 4 replicates.

Sample	SSL (days)	Variation in TTP (%)	Variation in TA (%)	Variation in PA (%)	Variation in CI (%)	Variation in T (%)	Variation in VA (%)
Y-N	3	-1.54 ± 0.23	-2.98 ± 0.15	-1.29 ± 0.31	-1.71 ± 0.14	1.74 ± 0.21	2.41 ± 0.19
	7	-3.03 ± 0.18	-7.47 ± 0.25	-2.87 ± 0.30	-8.53 ± 0.17	4.41 ± 0.29	6.16 ± 0.13
	15	-12.16 ± 0.11	-16.62 ± 0.19	-11.52 ± 0.29	-17.78 ± 0.16	12.16 ± 0.34	10.64 ± 0.25
	30	-17.03 ± 0.13	-21.70 ± 0.23	-17.17 ± 0.31	-22.26 ± 0.47	18.97 ± 0.31	17.02 ± 0.24
Y-P	3	-0.33 ± 0.16	-1.20 ± 0.17	-0.90 ± 0.33	-3.05 ± 0.33	1.26 ± 0.27	1.16 ±0.17
	7	-3.31 ± 0.22	-7.99 ± 0.32	-3.82 ± 0.27	-5.43 ± 0.13	3.36 ± 0.33	4.81 ± 0.16
	15	-6.60 ± 0.33	-9.02 ± 0.43	-7.47 ± 0.34	-10.47 ± 0.45	7.26 ± 0.34	7.12 ± 0.12
	30	-8.83 ± 0.46	-11.31 ± 0.26	-10.72 ± 0.42	-15.12 ± 0.32	10.84 ± 0.22	8.07 ± 0.13
Y-C	3	-4.24 ± 0.27	-5.57 ± 0.17	-4.06 ± 0.31	-4.24 ± 0.17	3.31 ± 0.37	3.16 ± 0.17
	7	-9.08 ± 0.25	-14.11 ± 0.15	-9.34 ± 0.17	-6.97 ± 0.37	7.49 ± 0.31	9.56 ± 0.25
	15	-14.45 ± 0.21	-19.99 ± 0.21	-14.57 ± 0.27	-18.61 ± 0.24	12.16 ± 0.47	10.86 ± 0.19
	30	-18.79 ± 0.41	-26.09 ± 0.41	-20.12 ± 0.17	-24.00 ± 0.35	19.90 ± 0.17	16.69 ± 0.43
Y-S	3	-2.15 ± 0.23	-2.43 ± 0.27	-1.63 ± 0.20	-3.29 ± 0.23	1.60 ± 0.20	2.22 ± 0.11
	7	-9.10 ± 0.65	-5.68 ± 0.15	-7.89 ± 0.31	-6.21 ± 0.16	4.53 ± 0.31	8.99 ± 0.19
	15	-14.57 ± 0.35	-10.71 ± 0.25	-14.73 ± 0.30	-15.91 ± 0.45	12.15 ± 0.30	12.13 ± 0.29
	30	-17.76 ± 0.42	-16.79 ± 0.42	-17.77 ± 0.29	-21.01 ± 0.22	18.29 ± 0.29	15.64 ± 0.18
Y-TB	3	-4.05 ± 0.28	-2.80 ± 0.15	-1.63 ± 0.11	-5.41 ± 0.23	3.77 ± 0.22	3.17 ± 0.28
	7	-4.74 ± 0.15	-12.11 ± 0.25	-13.47 ± 0.22	-13.20 ± 0.26	9.40 ± 0.11	4.78 ± 0.20
	15	-12.02 ± 0.16	-16.55 ± 0.29	-16.32 ± 0.24	-19.41 ± 0.35	15.23 ± 0.24	11.39 ± 0.14
	30	-16.56 ± 0.18	-23.73 ± 0.38	-20.10 ± 0.26	-24.09 ± 0.31	19.13 ± 0.26	17.98 ± 0.24
S-N	3	-2.60 ± 0.23	-4.46 ± 0.23	-3.53 ± 0.11	-1.97 ± 0.18	2.37 ± 0.19	2.32 ± 0.16
	7	-5.92 ± 0.27	-11.47 ± 0.18	-6.15 ± 0.13	-4.87 ± 0.26	6.34 ± 0.26	7.73 ± 0.30
	15	-10.45 ± 0.31	-19.91 ± 0.26	-9.42 ± 0.25	-12.90 ± 0.23	11.29 ± 0.32	12.28 ± 0.29
	30	-15.86 ± 0.44	-27.37 ± 0.34	-14.29 ± 0.24	-19.07 ± 0.34	16.22 ± 0.33	15.59 ± 0.31
S-P	3	-0.99 ± 0.19	-2.83 ± 0.16	-1.82 ± 0.29	-0.66 ± 0.10	0.36 ± 0.27	1.24 ±0.39
	7	-2.78 ± 0.32	-5.29 ± 0.27	-6.62 ± 0.26	-2.07 ± 0.27	2.40 ± 0.21	2.66 ± 0.27
	15	-4.84 ± 0.23	-9.99 ± 0.22	-9.80 ± 0.12	-6.73 ± 0.22	5.75 ± 0.39	3.72 ± 0.34
	30	-6.74 ± 0.36	-13.64 ± 0.31	-11.32 ± 0.33	-10.97 ± 0.31	9.13 ± 0.45	5.67 ± 0.22
S-C	3	-1.24 ± 0.19	-3.82 ± 0.28	-1.68 ± 0.17	-2.78 ± 0.28	2.26 ± 0.25	2.73 ± 0.17
	7	-5.95 ± 0.15	-10.12 ± 0.21	-7.87 ± 0.25	-7.00 ± 0.21	5.09 ± 0.27	6.67 ± 0.17
	15	-10.89 ± 0.21	-17.95 ± 0.29	-11.38 ± 0.49	-13.10 ± 0.29	10.47 ± 0.29	10.14 ± 0.27
	30	-15.72 ± 0.11	-25.93 ± 0.36	-15.93 ± 0.13	-19.67 ± 0.36	16.80 ± 0.23	14.93 ± 0.17
S-S	3	-1.64 ± 0.13	-3.32 ± 0.23	-1.68 ± 0.11	-2.98 ± 0.23	1.16 ± 0.25	1.99 ± 0.13
	7	-5.77 ± 0.15	-8.90 ± 0.35	-7.42 ± 0.39	-7.13 ± 0.35	4.78 ± 0.31	4.82 ± 0.31
	15	-9.73 ± 0.35	-16.13 ± 0.39	-11.59 ± 0.29	-13.80 ± 0.39	11.43 ± 0.29	7.68 ± 0.30
	30	-13.04 ± 0.42	-24.48 ± 0.46	-16.83 ± 0.28	-18.92 ± 0.46	15.92 ± 0.38	13.12 ± 0.29
S-TB	3	-1.21 ± 0.13	-3.13 ± 0.17	-2.30 ± 0.26	-3.29 ± 0.29	3.65 ± 0.16	2.18 ± 0.33
	7	-5.16 ± 0.25	-7.62 ± 0.21	-7.18 ± 0.20	-7.00 ± 0.15	6.98 ± 0.20	6.11 ± 0.22
	15	-6.75 ± 0.16	-14.43 ± 0.39	-13.10 ± 0.24	-13.91 ± 0.21	11.85 ± 0.24	10.77 ± 0.24
	30	-14.88 ± 0.38	-24.88 ± 0.45	-17.42 ± 0.34	-19.67 ± 0.41	16.22 ± 0.34	15.92 ± 0.26

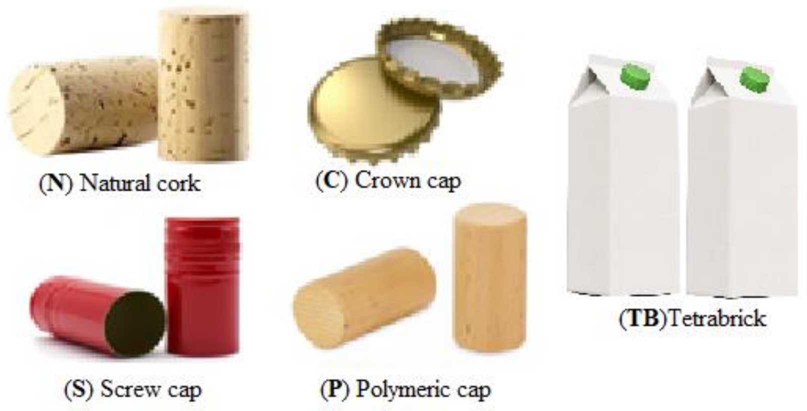

Figure S1. Image of the capping systems used in the trials.

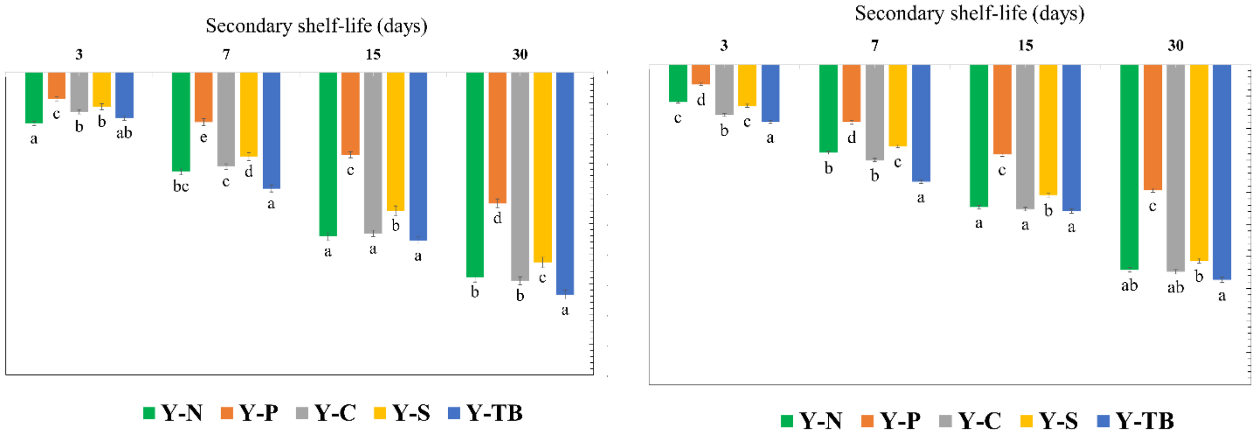

(**a**) (**b**)

Figure S2. Percentage decrease of SO_2 in young red wine (Y) during the Secondary shelf-life (3-7-15-30 days) in the test without air exchange (test 1). Data are expressed as mean ± SD for 4 replicates. Different letters in each group refer to significant differences (Tukey, $p \leq 0.05$): (**a**) Total SO_2 (**b**) Free SO_2.

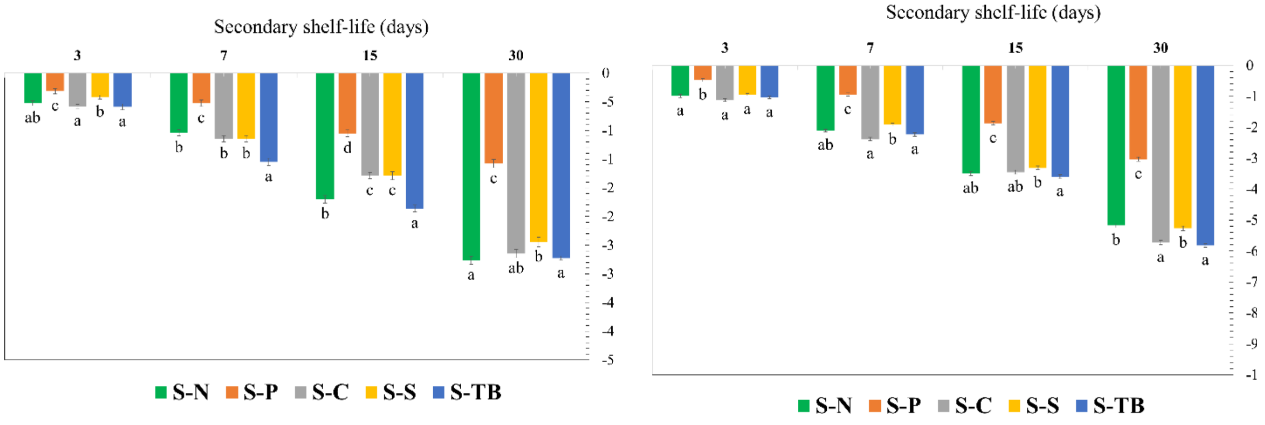

(**a**) (**b**)

Figure S3. Percentage decrease of SO_2 in structured red wine (S) during the Secondary shelf-life (3-7-15-30 days) in the test without air exchange (test 1). Data are expressed as mean ± SD for 4 replicates. Different letters in each group refer to significant differences (Tukey, p≤0.05): (**a**) Total SO_2 (**b**) Free SO_2.

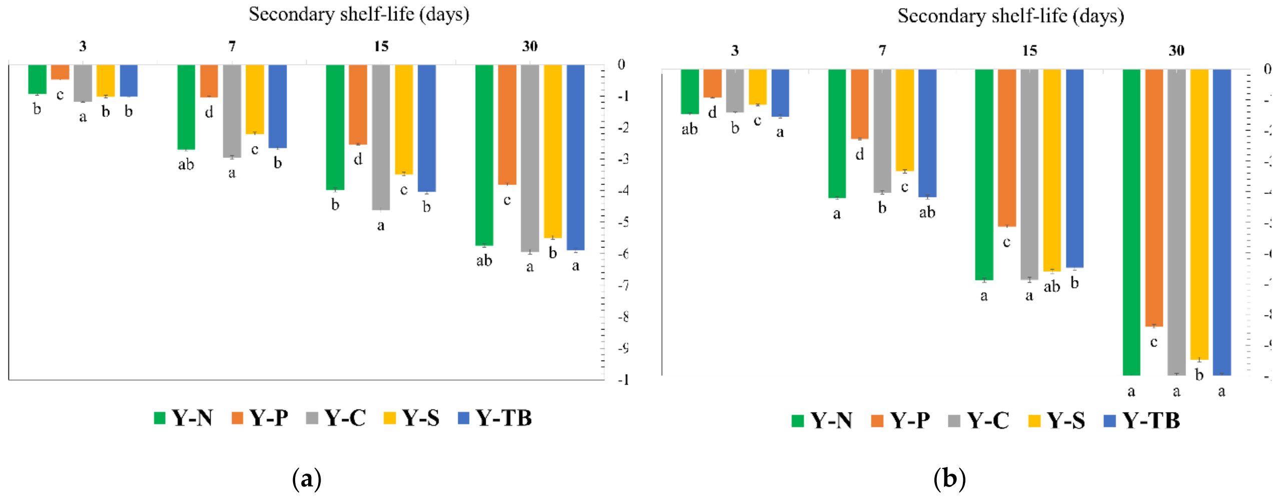

Figure S4. Percentage decrease of SO_2 in young red wine (Y) during the Secondary shelf-life (3-7-15-30 days) in the test with air exchange (test 2). Data are expressed as mean ± SD for 4 replicates. Different letters in each group refer to significant differences (Tukey, $p \leq 0.05$): (**a**) Total SO_2 (**b**) Free SO_2.

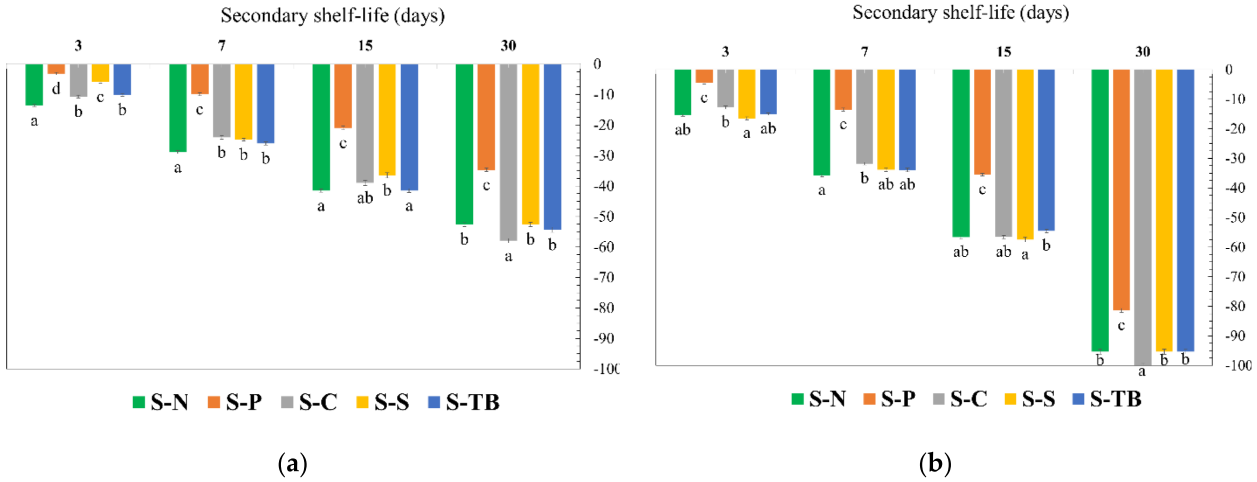

Figure S5. Percentage decrease of SO_2 in structured red wine (S) during the Secondary shelf-life (3-7-15-30 days) in the test with air exchange (test 2). Data are expressed as mean ± SD for 4 replicates. Different letters in each group refer to significant differences (Tukey, p≤0.05): (**a**) Total SO_2 (**b**) Free SO_2.

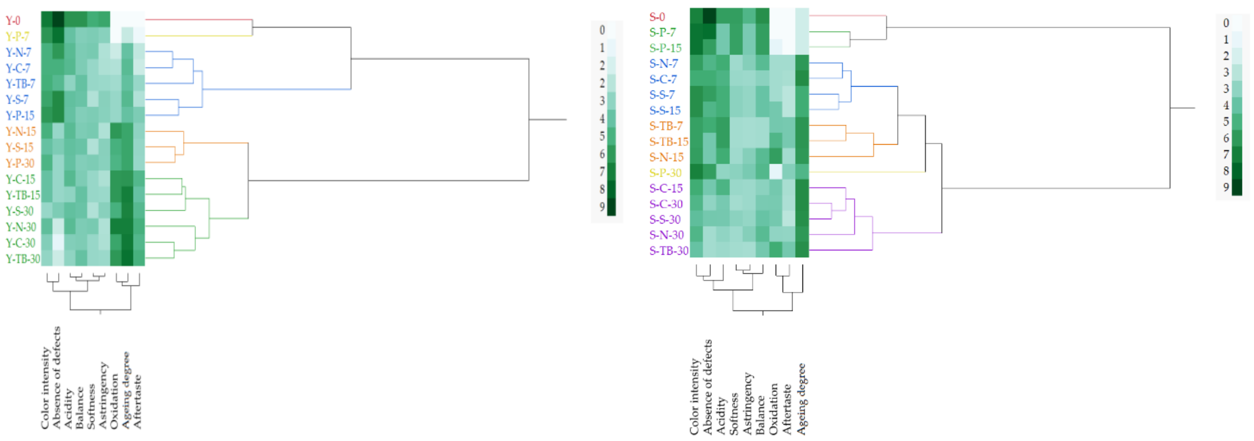

(**a**) (**b**)

Figure S6. Hierarchical cluster analysis (HCA) based on quantitative in the test without air exchange (test 1). The number next to the code (0-7-15-30) indicates the days of SSL for each sample: (**a**) Young red wine (**b**) Structured red wine.

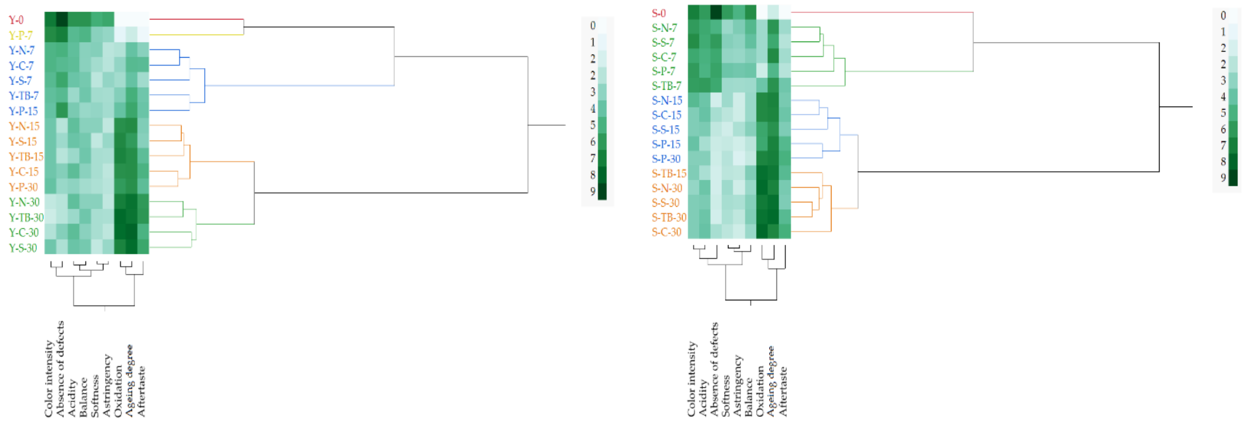

(**a**) (**b**)

Figure S7. Hierarchical cluster analysis (HCA) based on quantitative in the test with air exchange (test 2). The number next to the code (0-7-15-30) indicates the days of SSL for each sample: (**a**) Young red wine (**b**) Structured red wine.
